# Complete Genome Sequence of Achromobacter xylosoxidans Myophage Mano

**DOI:** 10.1128/MRA.01390-20

**Published:** 2021-01-14

**Authors:** Mariah Bartz, Guichun Yao, Tram Le, Mei Liu, Ben Burrowes, Carlos Gonzalez, Ryland Young

**Affiliations:** a Department of Biochemistry and Biophysics, Texas A&M University, College Station, Texas, USA; b Center for Phage Technology, Texas A&M University, College Station, Texas, USA; Loyola University Chicago

## Abstract

*Achromobacter* spp. are ubiquitous Gram-negative bacteria, some of which can cause respiratory tract infections in patients with autoimmune disorders and cystic fibrosis. Bacteriophages have therapeutic and biotechnological potential to combat *Achromobacter* sp. infections. This announcement details the 42.5-kb genome sequence of the temperate Achromobacter xylosoxidans myophage Mano.

## ANNOUNCEMENT

*Achromobacter* spp. are Gram-negative rod-shaped bacteria found throughout nature that can cause chronic, antibiotic-resistant infections in cystic fibrosis patients (1). Phage Mano could be adapted for use as a possible therapeutic or diagnostic agent for patients who have developed a refractory respiratory infection caused by *Achromobacter* spp.

Bacteriophage Mano was isolated by plaque purification (2) from a Michigan topsoil sample by plating on nutrient agar lawns of Achromobacter xylosoxidans grown at 37°C. Genomic DNA was purified using the Wizard DNA cleanup kit (Promega) as previously described (3) and prepared as Illumina TruSeq libraries. Sequencing was performed on an Illumina iSeq instrument with paired-end 2 × 150-bp reads using v2 500-cycle chemistry. The 241,262 raw sequence reads were quality controlled using FastQC v0.11.9 (www.bioinformatics.babraham.ac.uk/projects/fastqc) and manually trimmed using FASTX-Toolkit v0.0.14 (http://hannonlab.cshl.edu/fastx_toolkit/). The 42,452-bp genome was assembled using SPAdes v3.5.0 (4) with a resulting sequencing coverage of 229.2×. The genome was closed by PCR and Sanger sequencing of the product using the forward primer GCTTCGATATCTACGGTGCAAA and the reverse primer TTTCACCTGGATGGCCG. Structural annotations were performed using GLIMMER v3 (5) and MetaGeneAnnotator v1.0 (6). ARAGORN v5.33 was used for detection of tRNA genes (7). Gene function was predicted using InterProScan v5.22 (8), TMHMM v2.0 (9), HHPred (10), and BLAST v2.9.0 (11) against the NCBI nonredundant (nr), Swiss-Prot, and TrEMBL databases (12) with default settings. The annotation tools were used in the Galaxy and Apollo instances hosted at https://cpt.tamu.edu/galaxy-pub (13–15). Default parameters were used for all tools.

Mano was found to have 1 tRNA gene, 64 predicted protein-coding genes, a coding density of 97%, and a GC content of 64%, very close to the 65% of its host. Mano has *cos* termini, as determined by PhageTerm (16), and appears to be novel, being most closely related to *Pseudomonas* phage PPpW-3 (GenBank accession number NC_023006.1; 30% nucleotide identity [BLASTn]; 40 similar proteins [BLASTp]). All 11 predicted tail and tail fiber proteins (but no other proteins) are similar to the equivalent proteins of the paradigm temperate Escherichia coli myophage P2 (NC_001895.1). Phage Mano has a predicted lysis cassette that includes genes for an endolysin, two class I holin/antiholins, and outer and inner membrane spanin subunits. As expected for a P2-related phage, the Mano genome sequence encodes a predicted integrase, indicating a temperate life cycle. The single tRNA gene is located in a 200-bp intergenic region immediately upstream of the integrase gene and appears to be intact, but its position near the integrase and on the strand complementary to both flanking genes suggests that it is used as an attachment site for lysogenic integration.

### Data availability.

The genome sequence of Mano can be found via GenBank accession number MT708550.1. The associated BioProject, SRA, and BioSample accession numbers are PRJNA222858, SRR11558347, and SAMN14609642, respectively.
